# The microbiome associated with *Trichodorus primitivus* is enriched with *Janthinobacterium* compared to soil

**DOI:** 10.2478/jofnem-2025-0043

**Published:** 2025-09-26

**Authors:** Roy Neilson, Dale King, Maddy E. Giles

**Affiliations:** Ecological Sciences Department, The James Hutton Institute, Dundee DD2 5DA, Scotland, UK

**Keywords:** biodiversity, microbial ecology, proteobacteria, soil biota, soil ecology, *Xiphinematobacter*

## Abstract

Although soil biota mediates many key processes that deliver multiple environmental benefits, interactions between soil biota are not well characterized. In an ecological context, studies to date on the associations between nematodes and bacteria have mostly focused on either intracellular bacteria or bacteria that have a potential role in crop pathogenesis by endoparasitic nematode species, that is, those species that have a component of their life cycle within the plant host. Moreover, evolutionary studies have utilized the model nematode species, *Caenorhabditis elegans*, for studies on survival, behavior, and fecundity. In this study, we characterize the bacterial communities associated with an ectoparasitic nematode species, *Trichodorus primitivus*, whose complete life cycle is external to the plant host. Compared to the soil from which the nematodes were extracted, the diversity of bacterial communities associated with *T. primitivus* was reduced. By contrast, the nematode-associated bacterial community was significantly enriched with *Janthinobacterium*, a known antagonist of soilborne pathogens. This study advances knowledge on the interactions between bacteria and ectoparasitic nematodes, which could help inform the future development of novel strategies for nematode control.

Soil is multifunctional (Manning et al., 2018) with soil processes driven by soil biota (Bardgett and van der Putten, 2014). The interplay between soil biota is complex (Creamer et al., 2016), resulting in a broad spectrum of functional outcomes, for example, a modification of nutrient acquisition by plants (Mezeli et al., 2020) and soilborne pathogen management through suppressive soils (Schlatter et al., 2017). Yet, a detailed understanding of the roles and interactions of various soil taxa is only just emerging and remains mostly opaque (Thakur and Geisen, 2019).

Nematodes, one of the most abundant and diverse soil taxa (Hugot et al., 2001; van den Hoogen et al., 2019), and bacteria have close associations. For example, root herbivory by nematodes leads to an increase in total microbial biomass (Denton et al., 1998), antagonistic bacteria mitigate the effects of soilborne pathogenic nematode species (Elhady et al., 2017; Orr et al., 2020), microbial community size can be a potential predictor of nematode functional group (Neilson et al., 2020), and nematodes and bacteria operate in concert for environmental adaptation (Petersen et al., 2023). Furthermore, soilborne entomopathogenic nematode species harbor *Photorhabdus* and *Xenorhabdus* bacteria that cause intracellular death, resulting in insect host morbidity (Forst and Clarke, 2001). A few intracellular bacteria (Brown, 2018) have been infrequently reported to be associated with several (semi) endoparasitic plant-pathogenic nematode species (e.g., Walsh et al., 1983; Noel and Atibalentja, 2006; Denver et al., 2016; Showmaker et al., 2018; Wasala et al., 2019). Furthermore, intracellular bacteria belonging to *Xiphinematobacter*, a member of the *Verrucomicrobia*, have been reported from several ectoparasitic, that is, all life stages exterior to the plant host, plant-pathogenic nematode species belonging to the *Xiphinema americanum*-group (Vandekerckhove et al., 2000; Lazarova et al., 2016), and it has been postulated that *Xiphinematobacter* may confer functional benefit to the host nematode through essential amino acid enrichment (Myers et al., 2021).

Bacterial communities rather than selected individual intracellular bacterial species have been associated with a few (semi)endoparasitic nematode species, mostly in the context of either contributing to or ameliorating plant parasitism (e.g., Ladygina et al., 2009; Baquiran et al., 2013; Cao et al., 2015; Elhady et al., 2017, 2021; Topalović et al., 2019; Lamelas et al., 2020; Oro et al., 2020, 2021). Moreover, using the model bacterivorous nematode species *Caenorhabditis elegans* grown under laboratory conditions, a core microbiota was identified (Berg et al., 2016), and it was suggested that host rather than environmental factors shaped the bacterial communities. Similarly, using manipulative experiments with cultured bacterivorous nematode species *Acrobeloides maximus* and *C. elegans*, a core microbiome associated with *A. maximus* differed from both *C. elegans* and bulk soil (Baquiran et al., 2013). However, few studies have compared nematode-associated bacterial communities with those occurring in the bulk or rhizosphere soil environment (Ladygina et al., 2009; Shokoohi et al., 2022) and among several nematode feeding types, only one study (Ladygina et al., 2009) included an ectoparasitic nematode species (*Tylenchorhynchus* sp.). Thus, a clear knowledge gap exists regarding the relationship between bacterial communities of field populations of ectoparasitic nematode and rhizosphere/bulk soil.

Pathogenic soilborne nematodes, which agronomically are typically managed through chemical intervention, are major biotic stressors to global crop production, resulting in >10% yield losses with annual costs estimated at >US$125 billion (Chitwood, 2003). However, the move toward integrated pest management (Hillocks, 2012) and agroecological/regenerative practices (Puissant et al., 2021) requires a deeper understanding of interactions between nematodes and other soilborne taxa for their effective management. Members of the Trichodoridae are cosmopolitan ectoparasitic root-feeding nematodes. Approximately 120 Trichodoridae species (Decraemer et al., 2019; Xu and Zhao, 2019) are known, comprising two main genera, *Paratrichodorus* and *Trichodorus*, of which 13 species are known vectors of tobraviruses, including tobacco rattle virus (TRV), a viral pathogen of cultivated potato (*Solanum tuberosum*) (Taylor and Brown, 1997).

As Trichodoridae are economically important and their feeding mechanism is well characterized compared to most other ectoparasitic nematode groups, it is a good choice to characterize the bacterial community association between an ectoparasitic nematode species and the soil they inhabit. Based on previous studies, we hypothesize that the bacterial community associated with *Trichodorus primitivus*, a known virus-vector trichodorid species (Brown et al., 1989), is different from that of the soil they inhabit.

## Materials and Methods

### Nematode extraction

Soil samples were submitted by customers to the commercial diagnostic service managed by the lead author for species assessment of the free-living nematode community. Samples were from three fields (referred to herein as Fields 244, 301, and 423), each with humus-iron podzol as the dominant soil type. All fields were located in the major seed and ware potato growing regions of Perth and Kinross, in central Scotland. For commercial confidentiality and to comply with data protection regulations, it is not possible to provide detailed locations of the three fields. However, with the agreement of the individual farmers, fresh soil samples can be provided on request. Each field had what is considered locally a high abundance of *T. primitivus* (>5/g soil).

Soils were sampled to a depth of 10 cm using a grass plot sampler (internal diameter 2.3 cm, Eijkelkamp, Giesbeek, The Netherlands). A 1.5 kg composite soil sample was collected from each field, with each composite sample consisting of approximately 24 cores taken randomly along a standard “W” pattern (Marshall et al., 1998). Soil samples were stored overnight at 4°C. Nematodes were extracted from a 200 g subsample of each soil (Wiesel et al., 2015) using a modified Baermann funnel method (Brown and Boag, 1988). After ca. 48 hr, extracted nematodes were collected in 20 ml of water and left to settle for ca. 2 hr. Thereafter, water was decanted to leave ca. 2 ml volume.

Each sample was placed under a low-powered binocular microscope (Wild, Germany) under ×30 magnification using ×20 eyepieces. To mitigate potential variability of bacterial communities at the level of individual nematodes, for each soil sample, 5, 10, 20, and 50 individual *T. primitivus* nematodes of mixed development stages, adults where possible, were hand-picked using a needle into 2 ml Eppendorf tubes containing ddH_2_O. As with the study of Baquiran et al. (2013), to capture all types (or as many as possible) of nematode-bacterial symbiotic associations, we did not surface-sterilize the worms after extraction from soil but simply washed them with double-distilled water to remove the loosely associated soil microorganisms. Each numerical nematode grouping was replicated five times. Thus, the study comprised a total of 60 samples.

### DNA extraction and sequencing

Once hand-picked, nematodes were freeze-dried and DNA extracted using a standard protocol of bead-beating (Donn et al., 2012) coupled with a PureLink Genomic DNA extraction kit (Invitrogen) according to the manufacturer’s instructions. Soil microbial DNA was extracted using a DNeasy PowerSoil Pro extraction kit (Qiagen) from 0.25 g of soil from each of the composite field samples. The resulting DNA extracts were used to create 16S rRNA gene amplicon libraries of the V4 hypervariable region. To produce sufficient PCR product for sequencing, two rounds of the following PCR conditions were used: 95°C for 3 min then 25 cycles of 95°C for 30 sec, 55°C for 30 sec, 72°C for 30 sec before a final elongation at 72°C for 5 min. The following primers were used 515F-Y GTGYCAGCMGCCGCGGTAA (Parada et al., 2016) and 806R GGACTACNVGGGTWTCTAAT (Walters et al., 2015). All PCRs were performed in triplicate with a negative control, visualized on an agarose gel, and pooled prior to clean up with Ampure beads (Beckman Coulter). Nextera XT indexes (Illumina) were used for the indexing step following the manufacturer’s instructions. All samples were pooled in equimolar amounts, quality controlled using a Qbit, and sequenced by the James Hutton Institute sequencing unit using an Illumina Miseq (2 × 250 bp). Sequences were submitted to the European Nucleotide Archive under the accession number PRJEB61056.

### Statistical analysis and bioinformatics

A total of 2,700,047 reads were obtained. Chimera removal, dereplication, pairing, and amplicon sequence variant (ASV) assignment were carried out using DADA2 in the Qiime2 environment (Bolyen et al., 2019), resulting in an average of 32,927 reads per sample once reads were paired. On average, 80% of sequences were paired and retained. The naïve-Bayesian classifier function, q2-feature classifier, and Silva database v138.1 (Quast et al., 2013) were used to assign taxonomy to ASVs. Singleton sequences were removed from any subsequent analysis, as were samples with <10,000 sequences. This resulted in the removal of two samples from downstream analysis.

For analysis of alpha diversity, samples were rarefied to 10,000 sequences using the *rrarefy* function in the R package vegan (Oksanen et al., 2024) within the R statistical environment. Species richness, species evenness, and Shannon and Simpson diversity metrics were calculated on rarefied data using *vegan*. Differences in diversity metrics were determined using ANOVA and a Tukey HSD post hoc test.

For beta diversity, relative abundance data were calculated, log transformed, and a distance matrix was calculated using an Alternative Gower transformation. Principal coordinate analysis (PCoA) was used to visualize beta diversity and differences in bacterial communities derived from soils and nematodes tested using PERMANOVA. Differences in the relative abundance of different bacterial phyla were assessed using a Kruskal–Wallis test.

Differential abundance on testing non-normalized sequence data was used to determine differences in ASV abundance between soil and nematode samples. This was performed using DeSeq2 (Love et al., 2014) and visualized by plotting log2fold change of differentially abundant taxa.

## Results

The number of nematodes used to characterize associated bacterial communities had a limited effect on the number of bacterial sequences obtained. There was a significant difference (*P* < 0.05) between the number of bacterial sequences obtained from 10 and 20 nematodes, an average of 22,995 vs 26,283 sequences, respectively, but no difference in bacterial sequence number from 5 and 20 nematodes, 5 and 50 nematodes, or 20 and 50 nematodes. Furthermore, the field from which nematodes were extracted and the interaction between the field and the number of nematodes did not impact the number of bacterial sequences obtained (data not shown).

**Figure 1:**
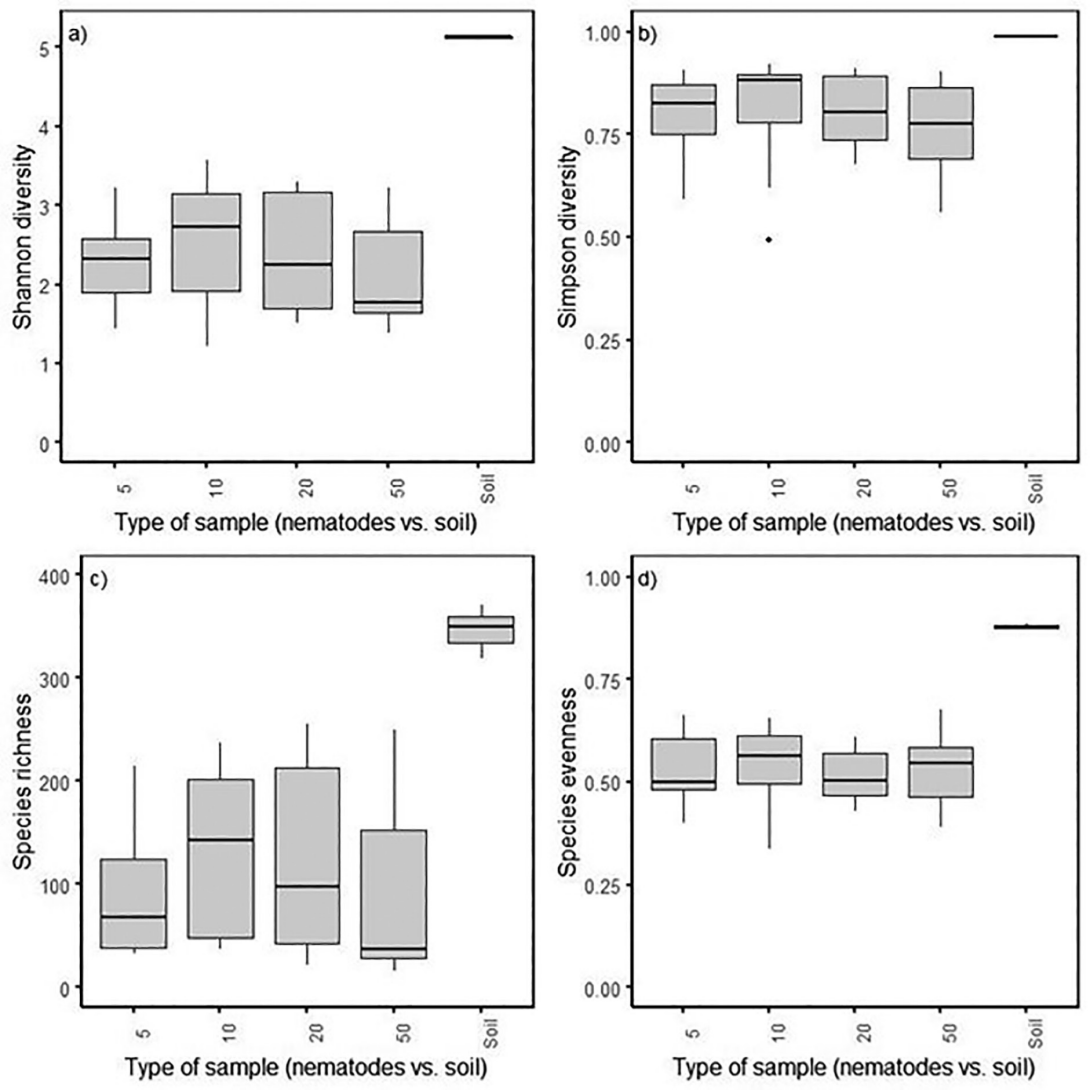
Boxplot of (A) Shannon diversity, (B) Simpson diversity, (C) evenness, and (D) species richness, for bacterial communities associated with *Trichodorus primitivus* nematodes and soil.

For alpha diversity, Shannon (*P* < 0.001), but not Simpson diversity of bacterial communities, differed between soil and nematode samples (Figs. 1A,B) and among fields (*P* < 0.05) but not by the number of nematodes extracted (Figs. S1A,B in Supplementary Materials). Similarly, species evenness (*P* < 0.001) and species richness (*P* < 0.001) of bacterial communities varied between soil and nematodes but not among fields or by the number of nematodes extracted (Figs. 1C,D; Figs. S1C,D in Supplementary Materials).

**Figure 2:**
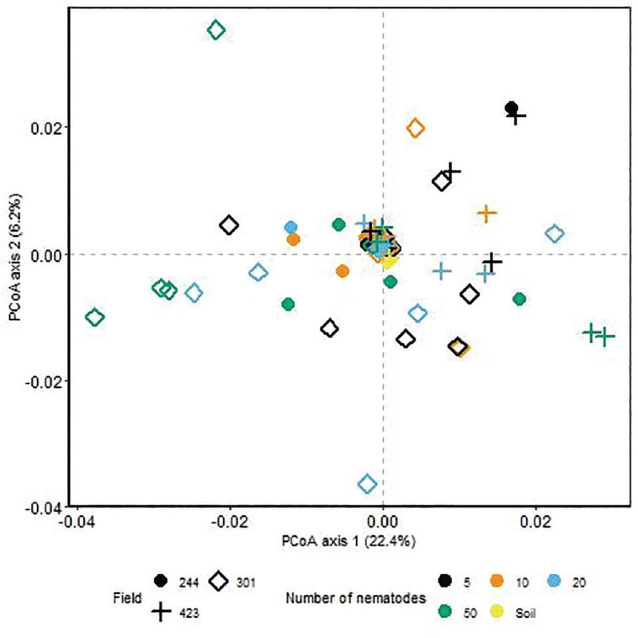
PCoA using Alternative Gower distance matrix with log_10_ relative abundances. Color denotes the number of *Trichodorus primitivus* nematodes, and shape denotes the sampled field. PCoA, principal coordinate analysis.

**Figure 3:**
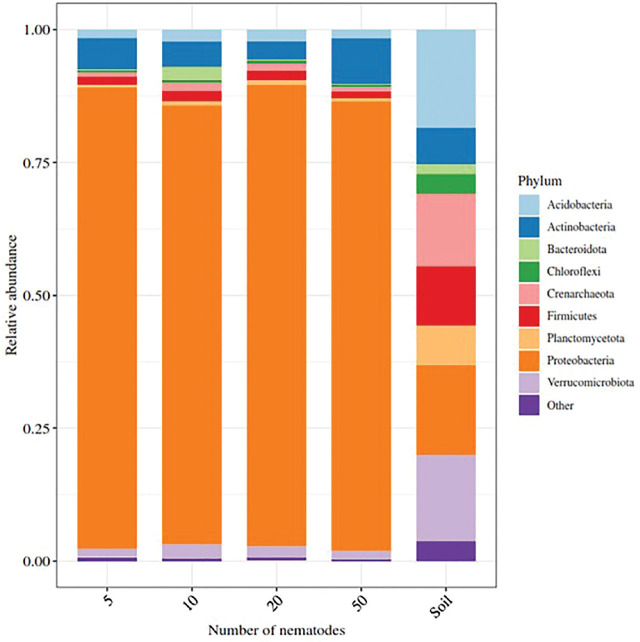
Stacked barplot of the relative abundance of bacteria at the phylum level associated with differing numbers of hand-picked *Trichodorus primitivus* nematodes and soil (all fields combined) from which the nematodes were extracted.

Overall, bacterial beta diversity differed between soil and nematodes (*P* = 0.003, Fig. 2). As with alpha diversity, there was no effect of nematode number or field on the bacterial beta diversity (Fig. 2). Reduced diversity and evenness of bacterial communities associated with nematodes was reflected in the dominance of Proteobacteria compared to soil bacterial communities (Fig. 3) across all sampled fields (Fig. S2 in Supplementary Materials).

**Figure 4:**
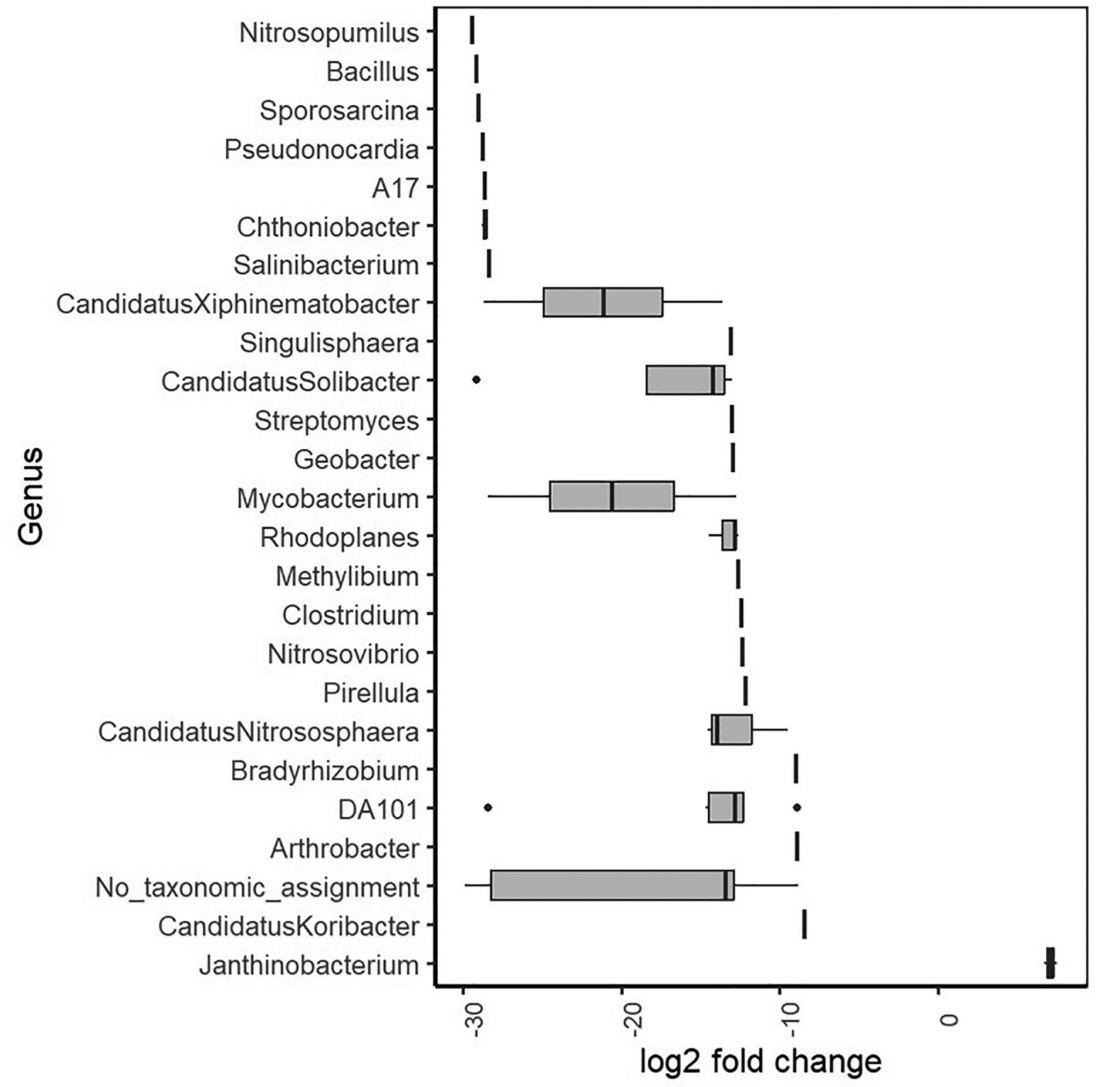
Log2 fold change in bacterial genera found to be differentially abundant (all fields combined). Values <0 are enriched in soil, and values >0 are enriched in nematodes.

A total of 114 ASVs were found to be differentially abundant between soil and nematode samples, including 19 ASVs from the phylum *Proteobacteria*, 19 from *Planctomycetes*, 18 from each of *Acidobacteria* and *Verrucomicrobia*, and 10 from *Actinobacteria*, the majority of which were enriched in soil compared to the nematode, *T. primitivus.* However, there was a significant enhancement of *Proteobacteria* abundance associated with nematodes compared to soil (*P* < 0.05), clearly driven by *Janthinobacterium* (Fig. 4).

## Discussion

The diversity of bacterial communities associated with the ectoparasitic nematode species, *T. primitivus*, was reduced compared to that of bacterial communities of the soil from which the nematodes were extracted. There is limited comparable published data, though using a cloning approach, it was reported that *Tylenchorhynchus* sp., also an ectoparasitic nematode species, had fewer OTUs (38 vs 163) and a lower Shannon diversity (3.0 vs 5.1) than the soil from which the nematodes were extracted (Ladygina et al., 2009). In the same study, this pattern was also reflected in bacterial communities associated with other nematode trophic groups (Ladygina et al., 2009). Bacterial communities associated with two free-living nematode species collected from the McMurdo Dry Valleys in Antarctica also exhibited reduced diversity compared to their surrounding environment (Parr McQueen et al., 2023).

In the present study, bacterial diversity did not differ between sampled fields, which differs from previous studies in the San Luis Valley (CO, USA) and Greece (Castillo et al., 2017; Boutsika et al., 2024) that, respectively, reported differences in bacterial community composition between geographically disparate fields. These contrasting results could be due to fields from the present study being in close geographic proximity (ca. 10 km), which perhaps has mitigated the strong biogeographic signal that typically perpetuates throughout soil microbiome studies (Bay et al., 2020). Alternatively, the similarity in multi-year crop rotations in the area, which are dominated by cereals, may have diluted any potential legacy effects of previous crops (Somenahally et al., 2018) or rhizodeposits (Nannipieri et al., 2023).

To date, most studies of bacterial communities associated with soilborne nematodes have been focused either on the role of bacteria in nematode pathogenesis of plants (Cao et al., 2015; Elhady et al., 2017, 2021; Lamelas et al., 2020), especially endoparasitic nematode species; the association of intracellular bacteria with a few ectoparasitic nematode species belonging to the *X. americanum*-group (Vandekerckhove et al., 2000, 2002; Denver et al., 2016; Lazarova et al., 2016; Orlando et al., 2016; Mobasseri et al., 2019; Myers et al., 2021; Palomares-Rius et al., 2021), of ecological associations (Martins do Rêgo Barros et al., 2025) or the use of antagonistic bacteria as a nature-based solution to manage pathogenic nematodes in production systems (Schlatter et al., 2017; Orr et al., 2020; Migunova and Sasanelli, 2021).

The dominance of Proteobacteria in bacterial communities associated with *T. primitivus* in this study compared with soil is consistent with that previously found for (semi)endoparasitic nematode species belonging to *Meloidogyne* and *Pratylenchus* (Adam et al., 2014; Cao et al., 2015; Elhady et al., 2017, 2021; Topalović et al., 2020, 2022, 2023; Yergaliyev et al., 2020); for the ectoparasitic species, *Tylenchorhynchus* sp. (Ladygina et al., 2009) and *Xiphinema elongatum* (Shokoohi and Masoko, 2024); and the omnivorous free-living species *Dorylaimus stagnalis* (Zheng et al., 2019). Among these studies, only Adam et al. (2014) reported *Janthinobacterium* as a dominant bacterial genus associated with their nematode of study, *Meloidogyne hapla*. However, it is noted that most of these studies used cultured rather than field-derived nematodes, and thus, unlike the present study, there may be a potential disconnect between the bacterial communities of nematodes and soil. Several members of the *Proteobacteria*, for example, *Burkholderia* and *Rickettsiales*, are considered to have a role in the nematode pathogenesis of crops, typically mitigating nematode infection through conferring suppressive actions (Adam et al., 2014; Cao et al., 2015; Elhady et al., 2017, 2021). Moreover, *Bradyrhizobium* has recently been reported as having nematicidal potential against *Meloidogyne incognita* (Sharma et al., 2025).

This assumed suppressive role for bacteria is a logical conclusion for (semi)endoparasitic nematode species that have a component of their life cycle within the plant host. However, *T. primitivus* feeding is enabled by an onchiostyle which perforates the root cells of host plants prior to ingestion of cell contents via a hollow feeding tube (Bird, 1971; Karanastasi et al., 2003, 2004) with the nematode always remaining external to the plant host. Thus, it is unclear whether there are similar derived benefits for the plant host through *T. primitivus* associations with the bacterial community, and particularly *Janthinobacterium*.

However, under controlled conditions, it has been reported that some strains of *Janthinobacterium* were toxic to the model nematode *C. elegans* (Swem et al., 2009; Hornung et al., 2013). Furthermore, *Janthinobacterium* has been reported to display broad antagonism against several soilborne pathogens, including *Pythium ultimum* and *Rhizoctonia solani* (Yin et al., 2021) and the fungal pathogen *Fusarium graminearum* (Haack et al., 2016). Consistent with *Janthinobacterium* having a pathogen suppressive role in soils, Xia et al. (2021) postulated that due to the enhanced abundance of *Janthinobacterium* in the rhizosphere of turfgrass, it facilitated nematode suppression. Thus, the snapshot measure in this study of a greatly enhanced abundance of *Janthinobacterium* associated with *T. primitivus* potentially reflects nematode suppressive activity. Alternatively, another possibility is that the bacteria have a role in nematode protection against predation, as has been previously reported for fungi (Büttner et al., 2021) and the model nematode, *C. elegans* (Haghani et al., 2024).

Unexpectedly, we identified seven ASVs associated with *T. primitivus* that were attributed to *Xiphinematobacter*, a well-characterized intracellular bacterium of *X. americanum*-group nematodes (Vandekerckhove et al., 2000, 2002; Lazarova et al., 2016; Orlando et al., 2016; Mobasseri et al., 2019). While in this study, the mean relative abundance of *Xiphinematobacter* was slightly greater in soil, the detection of *Xiphinematobacter* associated with *T. primitivus* perhaps represents a transient association between the bacterium and a nematode species phylogenetically disparate from the *X. americanum*-group (Donn et al., 2012). Moreover, the presence of *Xiphinematobacter* in soil in this study and as a dominant microbial taxon in a separate study of pristine grassland soils in southern England (Armbruster et al., 2021) challenges the current thinking that *Xiphinematobacter* is specific to nematode species belonging to the *X. americanum*-group. This is especially true given that *X. americanum*-group nematodes have to date not been recorded from the United Kingdom.

This study is not without constraints, including the process of extracting nematodes from soil (Parr McQueen et al., 2023), and we recognize that the number of fields sampled is low and that consequently this could potentially miss temporal and biogeographic variation, effects of (legacy) crop rotation (Elhady et al., 2021), and soil type (Castillo et al., 2017). However, this study advances knowledge on the interactions between bacteria and ectoparasitic nematodes, which could help inform the future development of novel strategies for nematode control (Petrushin et al., 2024).
